# Supplementary material for: Multispectral Depth-Resolved Fluorescence Lifetime Spectroscopy Using SPAD Array Detectors and Fiber Probes
Source: Sensors (Basel). 2019 Jun 13;19(12):2678. doi: 10.3390/s19122678 (PMC6631026; doi:10.3390/s19122678)
Supplement: Supplementary file 1 [file sensors-19-02678-s001.pdf]

Supplementary Materials

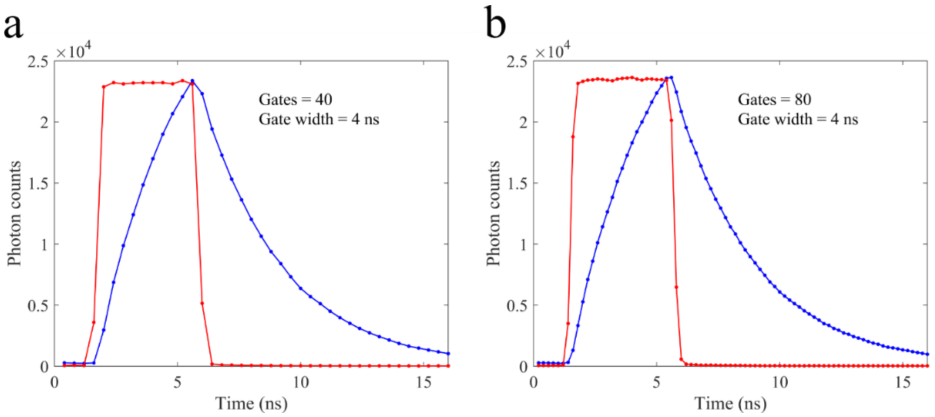

Figure S1. Representative instrument response function (in blue) and fluorescence intensity decays of fluorescence standards (in red) at different gate settings: (a) 40 sampling gates and 400 ps delay between gates; (b) 80 sampling gates and 200 ps delay between gates.

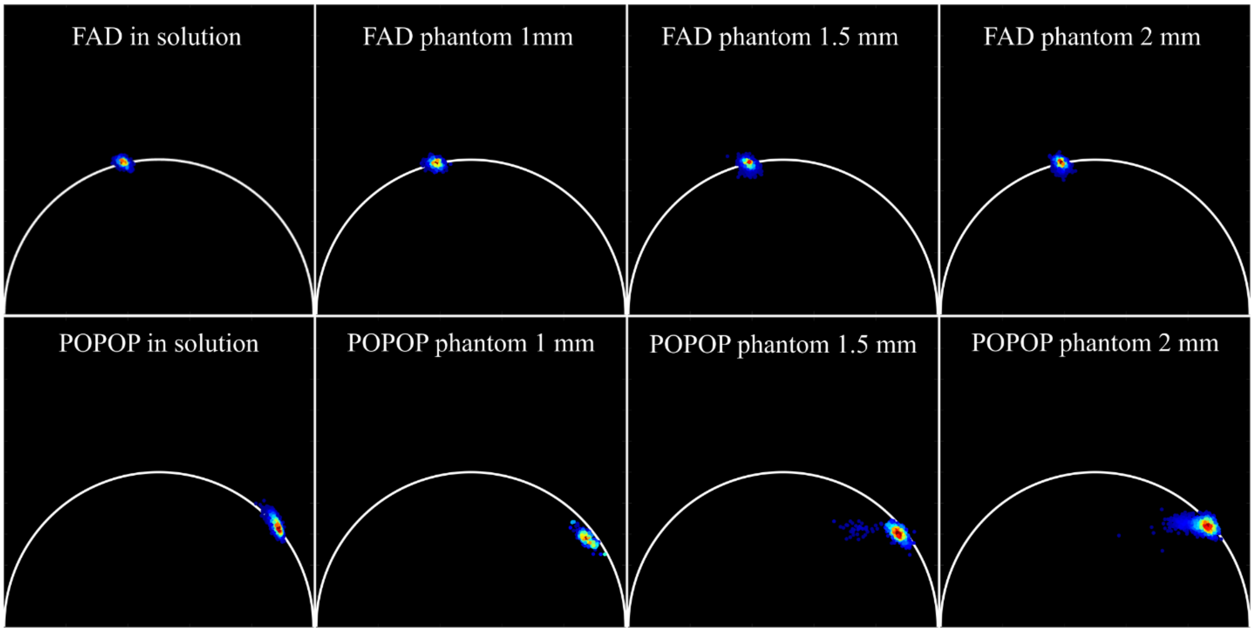

Figure S2. Fluorescence lifetime phasor maps of (top) FAD and (bottom) POPOP solutions and agarose phantoms.

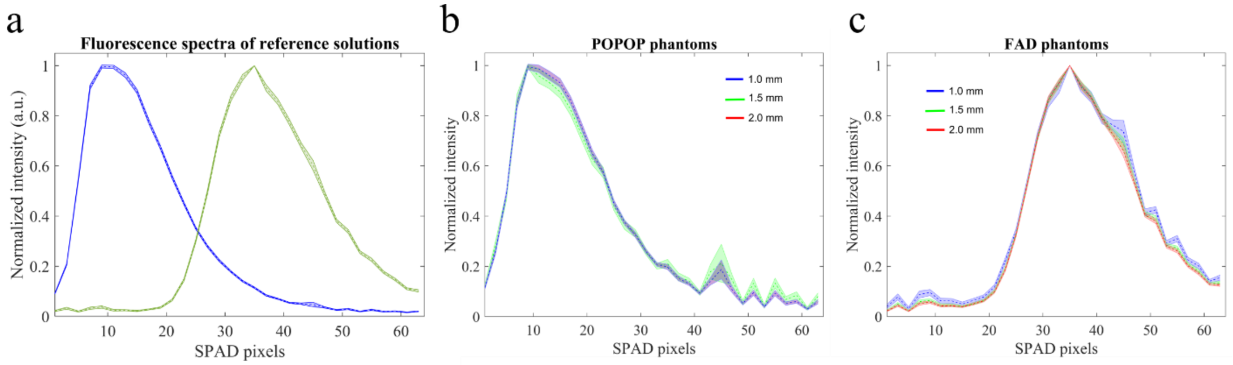

Figure S3. Fluorescence spectra of FAD and POPOP (a) solutions and (b, c) agarose phantoms.

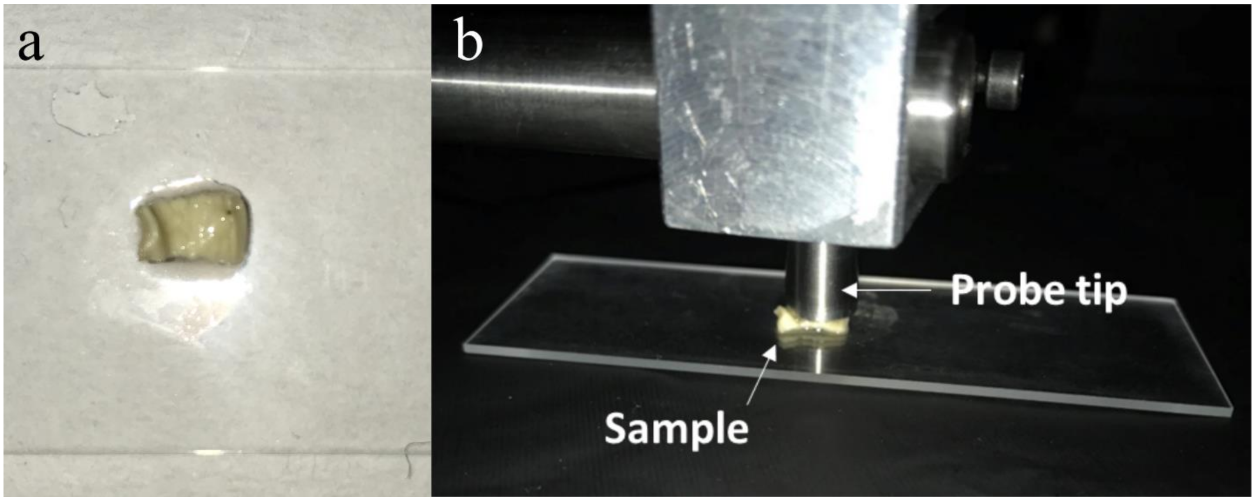

Figure S4. (a) Representative rabbit aorta sample on a glass slide; (b) Experimental setup with fiber tip in gentle contact with sample.
